# Reproducibility of quantitative indices of lung function and microstructure from ^129^Xe chemical shift saturation recovery (CSSR) MR spectroscopy

**DOI:** 10.1002/mrm.26310

**Published:** 2016-07-01

**Authors:** Neil J. Stewart, Felix C. Horn, Graham Norquay, Guilhem J. Collier, Denise P. Yates, Rod Lawson, Helen Marshall, Jim M. Wild

**Affiliations:** ^1^ POLARIS, Academic Unit of Radiology, University of SheffieldSheffieldUK; ^2^ Novartis Institutes for Biomedical ResearchCambridgeMassachusetts, USA; ^3^ Department of Respiratory MedicineSheffield Teaching Hospitals NHS Foundation TrustSheffieldUK

**Keywords:** hyperpolarized xenon‐129, lung function, chemical shift, reproducibility

## Abstract

**Purpose:**

To evaluate the reproducibility of indices of lung microstructure and function derived from ^129^Xe chemical shift saturation recovery (CSSR) spectroscopy in healthy volunteers and patients with chronic obstructive pulmonary disease (COPD), and to study the sensitivity of CSSR‐derived parameters to pulse sequence design and lung inflation level.

**Methods:**

Preliminary data were collected from five volunteers on three occasions, using two implementations of the CSSR sequence. Separately, three volunteers each underwent CSSR at three different lung inflation levels. After analysis of these preliminary data, five COPD patients were scanned on three separate days, and nine age‐matched volunteers were scanned three times on one day, to assess reproducibility.

**Results:**

CSSR‐derived alveolar septal thickness (ST) and surface‐area‐to‐volume (S/V) ratio values decreased with lung inflation level (*P* < 0.001; *P* = 0.057, respectively). Intra‐subject standard deviations of ST were lower than the previously measured differences between volunteers and subjects with interstitial lung disease. The mean coefficient of variation (CV) values of ST were 3.9 ± 1.9% and 6.0 ± 4.5% in volunteers and COPD patients, respectively, similar to CV values for whole‐lung carbon monoxide diffusing capacity. The mean CV of S/V in volunteers and patients was 14.1 ± 8.0% and 18.0 ± 19.3%, respectively.

**Conclusion:**

^129^Xe CSSR presents a reproducible method for estimation of alveolar septal thickness. Magn Reson Med 77:2107–2113, 2017. © 2016 The Authors Magnetic Resonance in Medicine published by Wiley Periodicals, Inc. on behalf of International Society for Magnetic Resonance in Medicine. This is an open access article under the terms of the Creative Commons Attribution License, which permits use, distribution and reproduction in any medium, provided the original work is properly cited.

## INTRODUCTION

Over the past two decades, hyperpolarized gas MRI with ^3^He and ^129^Xe has become a well‐established functional research tool for assessment of lung ventilation and microstructure 1, 2, 3. In recent years, a number of MR techniques have been developed to study pulmonary gas exchange with hyperpolarized ^129^Xe 4, 5, 6, exploiting the solubility of xenon in somatic substances and the large chemical shift difference of ^129^Xe in lung tissue and blood plasma (T/P) and red blood cells (RBCs) (corresponding to resonances at 197 and 218 ppm downfield from the ^129^Xe gas resonance, respectively). Notably, the chemical shift saturation recovery (CSSR) method 7, 8, 9 allows monitoring of gas exchange dynamics through acquisition of NMR spectra from the lungs at different delay times after selective saturation of the ^129^Xe magnetization in T/P and RBCs. This method has been shown to provide clinically relevant metrics of gas exchange impairment, enabling estimation of interstitial (septal) tissue thickening in interstitial lung disease (ILD), including idiopathic pulmonary fibrosis (IPF) 8, 10, and inflammation in chronic obstructive pulmonary disease (COPD) 11.

Previous studies with ^129^Xe CSSR in human subjects have been limited to small patient cohorts, and the reproducibility of the technique has yet to be assessed. The reproducibility of MR‐derived functional measures is critical to determining their sensitivity and robustness for future clinical applications as a quantitative outcome measure 12. For example, though the efficacy of ^3^He apparent diffusion coefficient mapping for characterization of emphysema has been well‐known for many years 13, the reproducibility of the technique is key to facilitating increased application in a clinical setting 14, 15. Similarly, ^129^Xe CSSR‐derived measures of pulmonary gas exchange must be demonstrated to be sufficiently reproducible before the sensitivity of the method to disease‐related changes in lung structure/function can be adequately assessed.

In this work, the intra‐subject reproducibility of ^129^Xe CSSR‐derived quantitative parameters of lung microstructure and function was evaluated in COPD patients and age‐matched healthy volunteers. Additionally, the sensitivity of the technique to MR pulse sequence strategy and to lung inflation state was examined by measuring the reproducibility of two existing implementations of the CSSR sequence, and performing CSSR experiments at different inflation levels in healthy volunteers, respectively.

## METHODS

This study was divided into two parts: (i) preliminary investigations of the reproducibility and robustness of performance of two different pulse sequence implementations of the CSSR method, and quantification of the effect of lung inflation level on CSSR‐derived parameters in healthy volunteers; and (ii) reproducibility measurements with one implementation of the CSSR sequence at fixed inflation level in COPD patients and age‐matched healthy volunteers.

**Figure 1:**
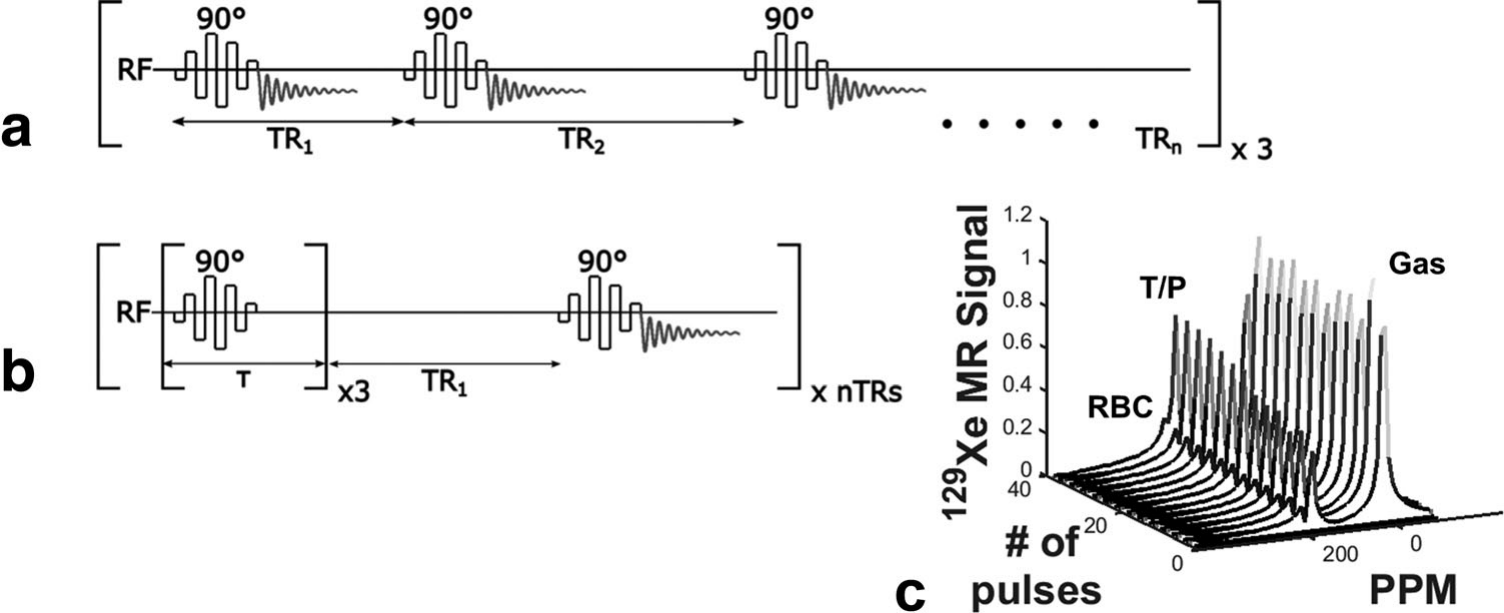
Schematic representation of the two implementations of the ^129^Xe CSSR pulse sequence used in this work: (a) 90 ° RF saturation pulses separated by a variable wait period (equivalent to the inter‐pulse repetition time); whole acquisition repeated three times (termed “multi‐sweep” CSSR); (b) multiple RF saturation pulses with a short inter‐pulse delay, followed by a variable wait period; whole time series acquired once (“multi‐sat” CSSR); (c) typical time series of ^129^Xe NMR spectra acquired using the sequence shown in (a), normalized to the gas peak. T/P, ^129^Xe dissolved in tissues and blood plasma; RBC, ^129^Xe in red blood cells.

Preliminary study (i): Five healthy volunteers (mean age ± standard deviation, 38 ± 14 years) with no history of respiratory disease were recruited (demographics given in Table 1). To examine the reproducibility of two different implementations of the CSSR sequence existing in the literature, each subject was scanned on three separate days over a period of 1–3 weeks on a 1.5 T GE HDx whole‐body MR system (GE Healthcare, Milwaukee, Wisconsin, USA). The two implementations of the ^129^Xe CSSR sequence are hereafter defined as the “multi‐sweep” and “multi‐sat” sequences (Fig. 1), referring to the use of multiple sweeps over different TR values, and multiple saturation pulses per TR interval, respectively. The multi‐sweep sequence uses single saturation pulses to destroy the magnetization of ^129^Xe dissolved in T/P and RBCs, followed by a variable inter‐pulse wait period (repetition time (TR)), during which alveolar‐capillary gas exchange occurs and polarized ^129^Xe gas diffuses into the T/P and RBC compartments. Multiple repeats of the TR sweep are acquired and averaged 10. The sequence parameters used were the same as in 10: binomial‐composite radiofrequency pulses were used for selective saturation of dissolved ^129^Xe 16; spectra were acquired with a bandwidth of 12 kHz and 64 sampling points; 25 TR settings from 20–1000 ms were swept through sequentially, and the whole TR sweep was repeated three times and averaged. The multi‐sat sequence, as detailed in 11, 17, employs multiple saturation pulses before each variable wait period and involves no averaging. This sequence was implemented with the following parameters: radiofrequency (RF) pulse and bandwidth as above; 128 sampling points (increased relative to the multi‐sweep implementation, because the minimum achievable exchange time is not limited by the read‐out duration in the multi‐sat sequence); and 21 TR values from 20–1000 ms. In each case, a gas dose of 350–400 mL xenon (86% ^129^Xe, polarized to ∼25% 18), balanced to 1 L with nitrogen, was inhaled from a Tedlar^®^ bag (Jensen Inert Products, Coral Springs, FL) from functional residual capacity (FRC) before a 10–15‐s breath‐hold. CSSR data were fitted with the model of xenon exchange (MOXE) 19, using a xenon diffusion coefficient in the dissolved phase of *D* = 3.0 x10^‐10^ m^2^s^‐1^ and an Ostwald solubility coefficient of xenon in tissue of 0.1 8, to estimate whole‐lung alveolar septal thickness (ST) and surface‐area‐to‐volume ratio (S/V).

**Table 1:** Subject Demographics, Pulmonary Function Tests, and Reproducibility of ^129^Xe CSSR ST and S/V Data.

Demographics		PFT parameters						MRI measurements			
Group	Age (sex)	FEV_1_ (% pred)	CV (%)	FEV_1_/FVC (% pred)	CV (%)	DL_CO_ (% pred)	CV (%)	Mean ST (µm)^a^	CV (%)	Mean S/V (cm^−1^)^a^	CV (%)
HV1	24 (M)	107.8	—	97.5	—	—	—	11.1 ± 0.3	2	286.7 ± 71.8	25
HV1	32 (F)	115.8	—	91.9	—	—	—	10.5 ± 0.5	5	259.9 ± 49.8	19
HV1	42 (M)	97.7	—	93.9	—	—	—	12.4 ± 0.7	5	237.7 ± 68.0	29
HV1	63 (F)	119.7	—	101.3	—	—	—	12.6 ± 0.6	5	171.8 ± 62.0	36
HV1	28 (M)	107.8	—	89.4	—	—	—	11.0 ± 0.4	3	186.3 ± 30.8	17
HV1	31 (M)	88.9	—	88.3	—	—	—	11.9	—	145.7	—
HV2	59 (F)	100.8	—	97.8	—	—	—	13.5 ± 0.4	3	164.2 ± 44.2	27
HV2	55 (M)	92.3	—	96.6	—	—	—	12.6 ± 0.3	3	238.6 ± 29.4	12
HV2	63 (M)	100.9	—	93.0	—	—	—	12.3 ± 0.7	6	154.7 ± 41.2	27
HV2	58 (M)	105.1	—	98.0	—	—	—	11.4 ± 0.6	5	261.8 ± 39.0	15
HV2	58 (F)	79.2	—	105.5	—	—	—	11.4 ± 0.4	4	180.4 ± 9.2	5
HV2	62 (M)	97.3	—	93.7	—	—	—	10.5 ± 0.1	1	185.5 ± 24.5	13
HV2	40 (F)	110.3	—	101.3	—	—	—	10.9 ± 0.6	5	84.4 ± 5.2	6
HV2	54 (M)	101.5	—	106.3	—	—	—	11.5 ± 0.3	3	322.8 ± 24.7	8
HV2	66 (M)	90.1	—	101.6	—	—	—	10.6 ± 0.7	7	155.9 ± 21.4	14
COPD	64 (F)	31.5 ± 0.7	2	33.2 ± 0.6	2	33.2 ± 2.1	6	14.3 ± 1.8	12	70.9 ± 9.4	13
COPD	67 (F)	45.4 ± 1.6	4	49.8 ± 0.7	1	98.4 ± 3.8	4	9.6 ± 0.3	3	215.8 ± 105.8	49
COPD	71 (M)	31.0 ± 1.3	4	27.6 ± 1.1	4	13.6 ± 2.2	16	14.1 ± 1.2	9	75.5 ± 1.8	2
COPD	76 (M)	43.5 ± 2.1	5	32.5 ± 0.8	3	29.6 ± 2.0	7	16.5 ± 0.3	2	90.5 ± 2.2	2
COPD	59 (F)	38.6 ± 2.5	6	42.8 ± 0.8	2	38.0 ± 4.1	11	15.7 ± 0.6	4	130.0 ± 29.9	23

HV1, healthy volunteers recruited for preliminary investigations; HV2, age‐matched healthy volunteers recruited for reproducibility tests; COPD, patients with chronic obstructive pulmonary disease recruited for reproducibility tests; PFT, pulmonary function test; FEV_1_, forced expiratory volume in one second; FVC, forced vital capacity; DL_CO_, diffusing capacity of carbon monoxide; ST, septal thickness; S/V, surface‐area‐to‐volume ratio; CV, coefficient of variation; % pred, pulmonary functional parameters expressed as a percentage of a predicted value, based on demographic factors such as age and height.

a All values derived from scans at FRC + 1 L using the multi‐sweep sequence.

To investigate the change in CSSR‐derived parameters with lung inflation level, multi‐sweep CSSR data were acquired at three different lung inflation levels from three healthy volunteers (24 (M), 28 (M), and 31 (M) in Table 1). Sequence parameters were as above. The following inflation levels were attained before the breath‐hold and data acquisition: i) forced exhalation to residual volume (RV), followed by inhalation of the 1 L xenon‐nitrogen mixture; ii) exhalation to FRC, followed by a 1 L inhalation; and iii) exhalation to FRC, followed by inhalation of the 1 L dose and additional inhalation of room air to reach total lung capacity (TLC).

Reproducibility measurements (ii): Nine healthy volunteers (59 ± 8 years) with no history of respiratory disease, and five patients with COPD (67 ± 6 years) were recruited (see Table 1). To assess the short‐ and long‐term reproducibility of ^129^Xe CSSR, COPD patients were scanned on four occasions; twice on the first day, once the following day, and once two weeks later. Volunteers were scanned in three separate sessions on the same day, between which they were repositioned. In all cases, the multi‐sweep sequence was employed, with parameters as previously and a xenon dosage of 300–350 mL.

For comparison with MR measurements, conventional pulmonary function tests (PFTs) were performed by all subjects (Table 1), including forced expiratory volume in 1 s (FEV_1_) and forced vital capacity (FVC) maneuvers. In addition, the diffusing capacity of carbon monoxide (DL_CO_) test was performed by COPD patients to provide a standard metric of pulmonary gas exchange. For the two healthy volunteer cohorts, PFT data were acquired on only one occasion, as the variability of spirometry is well‐known in healthy subjects 20, whereas for COPD patients, PFT data were obtained on each of the three scan dates.

To evaluate the reproducibility of each CSSR‐derived functional parameter, the intra‐subject standard deviation (SD) and coefficient of variation (CV; the ratio of SD to mean value, expressed as a percentage) was calculated. A mixed‐model, repeated measures analysis of variance test was performed for each parameter to determine the significance of any time‐dependent variations, according to the *F*‐value (analogous to a conventional statistical t‐value) and *P*‐value of significance. Reproducibility data are presented as mean, SD, and CV values and modified Bland‐Altman plots 21 with CV on the *y*‐axis. An equivalent analysis was carried out for PFT measurements where applicable.

## RESULTS

In one participant from the age‐matched healthy volunteer cohort (62 (M)), the signal‐to‐noise ratios (SNRs) of the spectra obtained from one scan were insufficient to fit meaningful estimates of alveolar ST and S/V. Additionally, another participant from this group (59 (F)) was unable to maintain breath‐hold for the duration of one scan. Hence, only two data points were used for reproducibility analysis in these subjects. For two COPD patients (64 (F) and 67 (F)), data were only successfully acquired once on the first day of scanning, and in a third patient (71 (M)), the spectral SNR was insufficient in one scan from the first day. Thus, only three of the four proposed acquisitions were available for reproducibility analysis in these patients.

**Figure 2:**
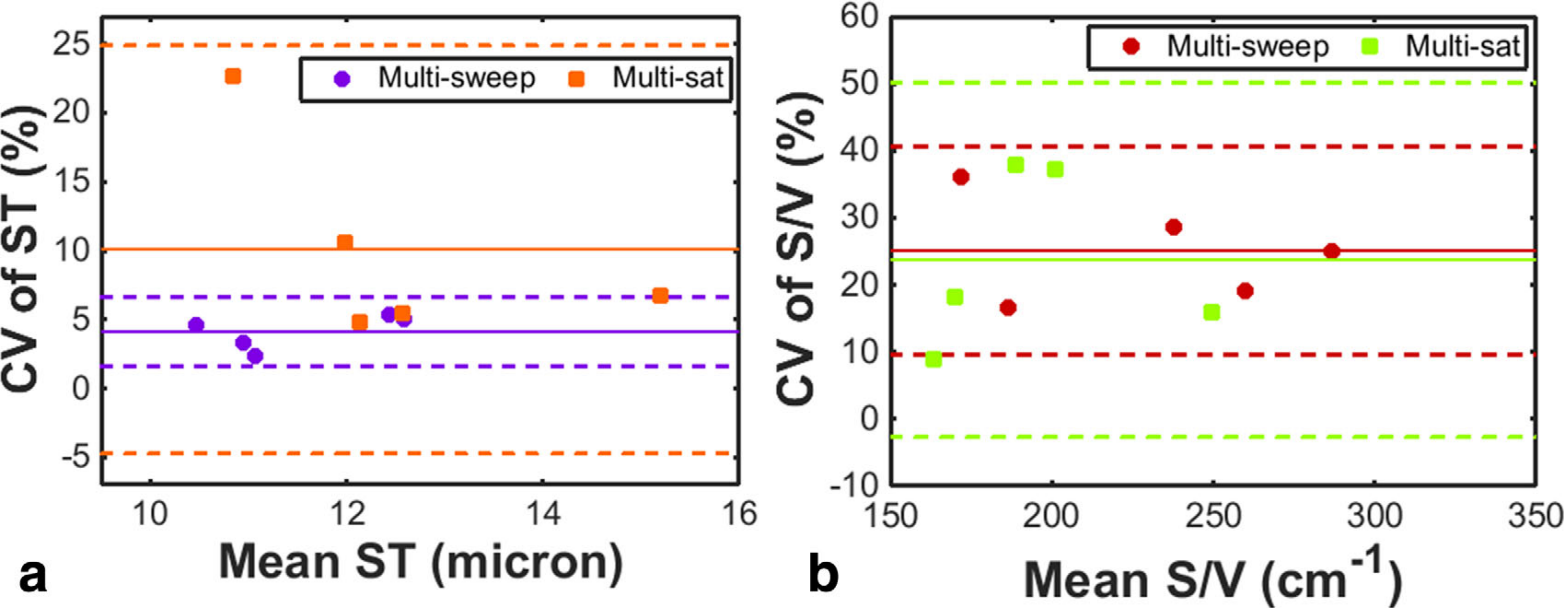
Preliminary investigation of the reproducibility of parameters derived from the two different implementations of the ^129^Xe CSSR sequence, as assessed in five healthy volunteers. The coefficient of variation of CSSR‐derived septal thickness and surface‐area‐to‐volume ratio values are plotted on Bland‐Altman charts in (a) and (b), respectively. Solid lines represent the mean CV and dashed lines denote ± 2 standard deviations from the mean.

Preliminary study (i): Mean ST values in the five healthy volunteers derived from multi‐sweep and multi‐sat sequences were 11.5 ± 0.9 µm and 12.6 ± 1.2 µm, and the corresponding mean CV values of ST were 4.1 ± 1.3% and 10.0 ± 7.4%, respectively. The mean S/V values derived from the multi‐sweep and multi‐sat sequences were 228 ± 49 cm^‐1^ and 195 ± 46 cm^−1^, and the corresponding mean CV values of S/V were 25.1 ± 7.8% and 23.7 ± 13.2%, respectively. Figure 2 depicts Bland‐Altman plots of the intra‐ and inter‐subject variations of ST and S/V parameters derived from the two sequences. (The intra‐subject variability (reproducibility) is represented by the CV axis.)

**Figure 3:**
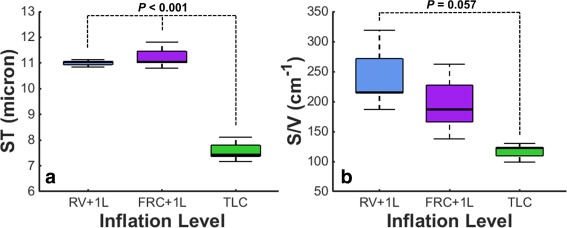
Mean ^129^Xe CSSR‐derived alveolar septal thickness (a) and alveolar surface‐area‐to‐volume ratio (b) measurements as a function of lung inflation level in three healthy volunteers. RV, residual volume; FRC, functional residual capacity; TLC, total lung capacity.

Mean ST values from the three healthy volunteers were similar at inflation levels of RV + 1 L (11.0 ± 0.1 µm) and FRC + 1 L (11.3 ± 0.5 µm), whereas the ST was significantly reduced (*P* < 0.001) at TLC (7.6 ± 0.5 µm) when compared with both RV + 1 L and FRC + 1 L, as shown in Figure 3a. S/V values exhibited a decreasing trend with inflation level (Fig. 3b), with a significance of *P* = 0.057 between TLC (115 ± 16 cm^−1^) and RV + 1 L (253 ± 66 cm^−1^) (S/V at FRC + 1 L was 200 ± 62 cm^−1^).

**Figure 4:**
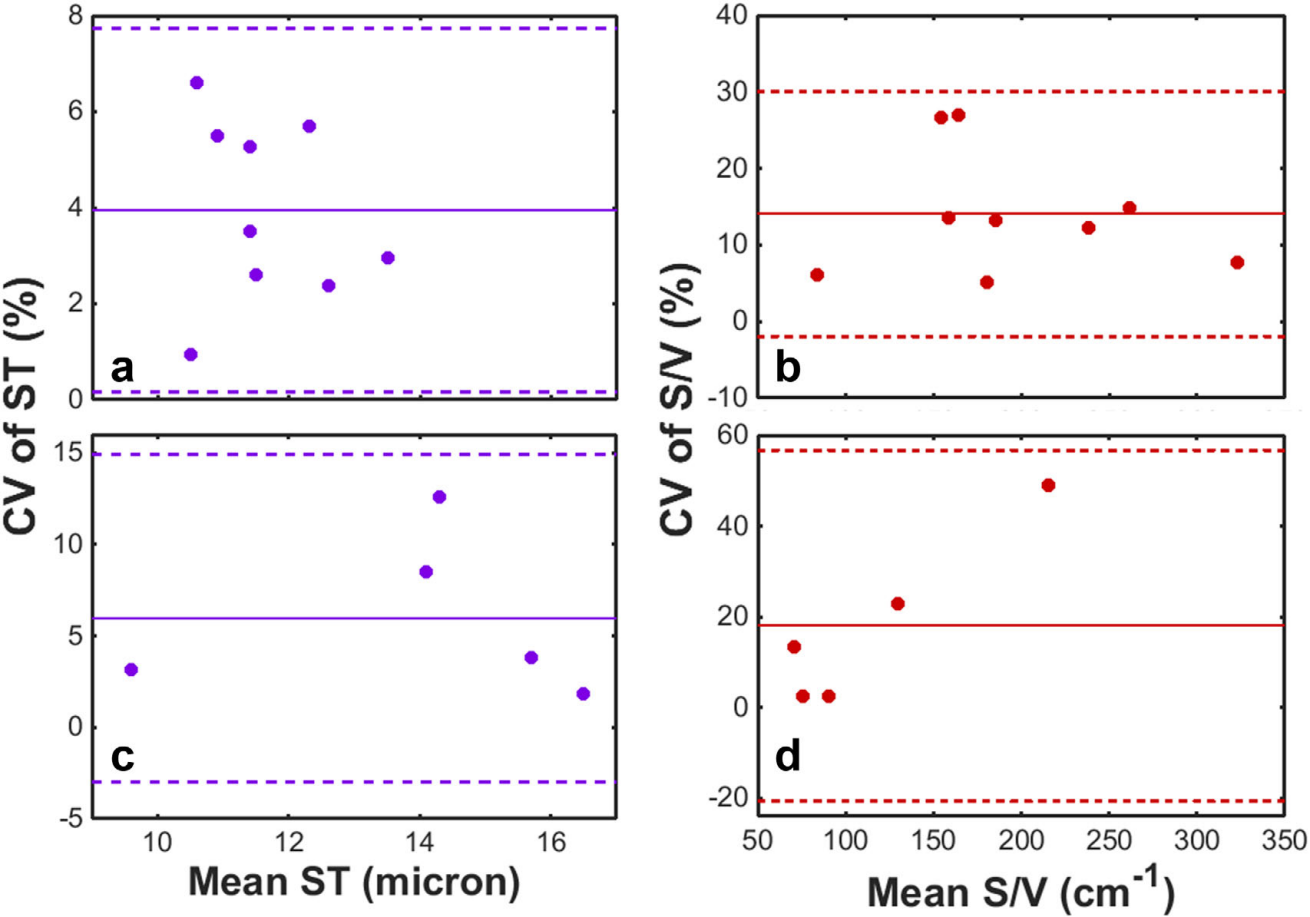
Reproducibility of ^129^Xe CSSR‐derived alveolar septal thickness and surface‐area‐to‐volume ratio values in COPD patients and age‐matched healthy volunteers. (a) and (b): ST and S/V values in healthy volunteers (mean age = 59 ± 8 years). (c) and (d): ST and S/V values in COPD patients (mean age = 67 ± 6 years). Solid lines represent the mean CV and dashed lines denote ± 2 standard deviations from the mean.

Reproducibility measurements (ii): The average of the mean ST in COPD patients (14.0 ± 2.7 µm) was elevated when compared with age‐matched healthy volunteers (11.6 ± 1.0 µm) (*P* < 0.05). Additionally, evidence of a reduced S/V ratio in COPD patients (117 ± 60 cm^−1^) when compared with volunteers (194 ± 70 cm^−1^) was observed (*P* = 0.055). The mixed‐model analyses showed no significant changes in ST or S/V as a function of scan time point, with *F* = 1.32, *P* = 0.294 and *F* = 2.48, *P* = 0.116 for healthy volunteers; *F* = 2.08, *P* = 0.156 and *F* = 0.27, *P* = 0.845 for COPD subjects, concerning ST and S/V, respectively. CV values of ST were <8% and <13% in volunteers and COPD patients, with a mean ± standard deviation of 3.9 ± 1.9% and 6.0 ± 4.5%, respectively (see Figs. 4a and 4c). CV values of S/V were < 28% and <50% in volunteers and COPD patients, with a mean ± standard deviation of 14.1 ± 8.0% and 18.0 ± 19.3% (Figs. 4b and 4d).

Average FEV_1_ and FEV_1_/FVC measurements were significantly reduced in COPD patients when compared with volunteers (*P* < 0.001) (Table 1). In patients, the mean CV values associated with pulmonary function tests were 4.2 ± 1.5% for FEV_1_; 2.4 ± 1.1% for FEV_1_/FVC; and 8.8 ± 4.8% for DL_CO_.

## DISCUSSION

The observation of improved reproducibility (lower CV) of ST for the multi‐sweep compared with the multi‐sat implementation of ^129^Xe CSSR may be a result of the reduction in variance of fitted data points arising from the multiple‐averaging process. Nevertheless, upon separate analysis of the individual TR sweeps of the multi‐sweep data sets presented in Figure 2, no clear difference in the mean CV of ST or S/V values derived from each of the sweeps—when compared with the multi‐sweep average—was observed, other than the CV of ST of the first sweep being slightly higher than that of the other two sweeps (mean CV of ST = 4.1 ± 1.3% for all sweeps, 5.5 ± 1.3% for sweep 1, 3.5 ± 2.4% for sweep 2, 3.7 ± 1.5% for sweep 3). We suspect that the minor increase in CV for the first sweep results from the fact that this sweep is associated with the highest SNR data‐points, and is thus more sensitive to subtle changes in the shape of the uptake curve between scans. The latter two sweeps have lower SNR, and hence we postulate that the fitting process results in a curve that approximates a noisier data set, such that subtle changes between repeated scans may be less apparent. Additionally, it is worth noting that the mean CV for the multi‐sat sequence is strongly influenced by an outlying data point with a CV of ST of 22.7%, which resulted from an anomalous ST value in one of the three repeated scans for that subject. In the absence of this outlier, the mean CV between the two implementations would be considerably closer (mean CV of multisat CSSR would become 6.9±2.6% instead of 10.0±7.4%). Previously, multiple saturation pulses have been required to completely destroy the dissolved‐phase ^129^Xe magnetization in human subjects 8, 11, 17. However, the use of a custom binomial‐composite pulse design permits highly effective saturation of magnetization with a single pulse 16. The observation that the CV of S/V is similar between the two sequence implementations implies that the saturation process itself is reproducible, as the S/V is strongly influenced by the early time points of the CSSR experiment, in which incomplete saturation could cause significant effects. In light of the apparent improved reproducibility of ST, the multi‐sweep implementation was employed for subsequent experiments.

The measured decrease in S/V and ST with lung inflation level can be explained by the expansion of the alveoli and stretching of the lung tissue at high inflation levels, respectively 22. A reduction in CSSR‐derived S/V has been previously reported with lung inflation level 23, and, although a change in CSSR‐derived ST with inflation level has not been reported, recent observations of a decline in the ratio of ^129^Xe T/P‐ or RBC‐to‐gas‐phase resonances with increasing inflation level 11, 24 indicate a reduced contribution of the dissolved ^129^Xe MR signal at higher inflation levels because of the lower volume fraction of tissue and capillaries versus airspace. Despite our observations, the interpretation of these data must be carefully considered in terms of the meaning of the different inflation levels. Although some recent ^129^Xe CSSR studies in humans have used the same approach as that described here to achieve a desired inflation level (e.g. FRC + 1 L) before data acquisition 17, others have employed a procedure of quantifying a subject's TLC before MRI, and modifying the inhaled gas dose for each subject to achieve an inflation level that equates to a specific fraction of that subject's TLC 8, 11. The latter procedure ensures that the lungs are at an equivalent level of inflation in each subject, which should correspond to a comparable alveolar geometry. The inflation level of FRC + 1 L employed here may equate to a different fraction of each subject's TLC. In future work, it may be better practice to calculate an inhaled gas dose such that the lung inflation level would be proportionate between subjects (e.g. 50% TLC), making comparisons among subjects more physiologically meaningful.

Assuming that the derived variation in ST and S/V with lung inflation level cannot be fully explained by inter‐subject differences in relative lung inflation level, this observation is an important consideration for future CSSR studies; it is crucial to carefully instruct the subject to ensure the desired inflation level is achieved and the breath‐hold is effectively maintained. It might be expected that patients would better tolerate breath‐holds at higher inflation levels than FRC + 1 L. Furthermore, we might expect the reproducibility of derived ST and S/V metrics at TLC to be improved when compared with FRC + 1 L, because TLC represents an extreme limit of lung inflation level, potentially easier to achieve for the patient, whereas the patients' perception of FRC may vary between experiments. However, appropriate reproducibility tests must be carried out at TLC before routine ^129^Xe CSSR scans are possible at that inflation level.

The mean ST and S/V values derived in this work are comparable to estimates obtained from alternative methods: ST ∼10 µm from histological methods 25 including computerized morphometry 26, 27; and S/V in healthy volunteers ∼250 cm^−1^ from histological methods 28 and 200–240 cm^−1^ from hyperpolarized ^3^He diffusion‐weighted MRI 29 (both of these articles also reported a reduced S/V of ∼50–150 cm^−1^ in a range of patients with severe to mild emphysema). In addition, our results are of the same order as those reported in recent ^129^Xe CSSR studies with human subjects 8, 10, 11, 17. However, direct comparison of the absolute ST and S/V values in this work and other ^129^Xe CSSR studies in humans requires careful consideration of any discrepancies in data acquisition and analysis approaches in those works, as discussed below. A review of example literature references for ST and S/V values and a detailed explanation of the challenges in directly comparing our values to those of other ^129^Xe CSSR studies in humans is included as supplementary text in the online version of this article.

It is important to consider that the data presented in this manuscript can be analyzed using models other than MOXE 8, 30, 31. In previous work, we highlighted small differences in CSSR‐derived lung microstructural parameters resulting from some of these models 10. Furthermore, in addition to discrepancies in data analysis procedure, most human studies with ^129^Xe CSSR to date have been performed with slightly different assumptions about the value of critical physical parameters 8, 10, 11, 17, 19, such as the diffusion coefficient and Ostwald solubility of xenon in tissue, both of which have a significant bearing on the absolute derived ST and S/V estimates. A review of the approaches and assumptions considered in each of these works is included as supplementary text and Supporting Table S1 in the online version of this article.

The mean intra‐subject standard deviation of CSSR‐derived ST values of age‐matched healthy volunteers (0.46 ± 0.21 µm) is much less than the difference in mean ST between healthy subjects and IPF patients (∼7 µm) as quantified previously (mean healthy subject ST = 10.0 ± 1.6 µm; mean IPF patient ST = 17.2 ± 1.1 µm from 10), and also less than the 2.4µm mean difference between healthy subjects and COPD patients as measured here. In addition, the Bland‐Altman plots in Figure 4 illustrate that the CV of ST does not appear to change with the mean value. These factors, coupled with the generally low CV values from volunteers and COPD patients (<8% and <13%, respectively), provide substantive evidence that the CSSR‐derived ST is a reproducible parameter. The CV of ST was comparable to that of PFT metrics of gas exchange; mean CV values in COPD patients were comparable to the reported long‐term variability of DL_CO_ (9% 32), whereas the CV in healthy volunteers was of a similar magnitude to that of same‐session variability of DL_CO_ 33.

In contrast, the mean CV values of S/V were above the target range for reproducibility of DL_CO_. This constrains the interpretation of apparent trends of reduced S/V in COPD patients and changes with lung inflation level. The S/V parameter is derived from the early time points of the CSSR experiment, when the dissolved ^129^Xe signal‐to‐noise ratio is lowest 7. In addition, it would be expected that incomplete saturation of magnetization would adversely affect the derived S/V more so than the ST, because the latter is predominantly influenced by later time points. Furthermore, the complexity of the model of xenon exchange 19 and its multiple interdependent fitting parameters may lead to inaccurate estimates of S/V (whereas by definition of the model, it could be expected that ST would be less influenced by these interdependencies). A combination of these factors may explain the relatively poor S/V reproducibility.

A further limitation of the reproducibility of the CSSR‐derived S/V in patients is the outlying data point (67 (F)), with 49% CV. This subject exhibited a lower ST than all subjects, and although her FEV_1_ and FEV_1_/FVC were < 50% of the predicted value, her DL_CO_ was 98.1% predicted, comparable to that of healthy subjects. Further study with larger patient populations and tighter lung inflation level control is necessary to identify these outliers and further validate the S/V reproducibility.

For both ST and S/V metrics, the intra‐subject reproducibility was noticeably worse in COPD patients than healthy volunteers, despite similar results obtained from the mixed‐model analysis. This may be partially explained by the fact that patients were scanned on multiple days, whereas volunteers were scanned on a single day. Because of poor‐quality or failed scans on the first day of the protocol, there was insufficient data available to accurately separate COPD patient reproducibility into same‐day or multi‐day reproducibility; hence, CV values for these patients are dominated by inter‐day variations. Additionally, it is worth considering that the reproducibility of the ^129^Xe CSSR measurement is dependent on a variety of factors, including fluctuations in ^129^Xe polarization, reproducibility of lung inflation level, and successful maintenance of breath‐hold for the scan duration; the latter two factors depend heavily on the subject. Patients may have difficulty in inhaling the complete contents of the Tedlar bag, and/or may be less effective in maintaining breath‐hold for the scan duration. It is possible to circumvent breath‐hold failure in the third sweep by analyzing data from the first or second sweeps only. However, factors such as lung inflation level are challenging to control, and it is prudent to carefully instruct the patient on the exact details of the protocol and perform training scans with bags of air before the CSSR scan itself, in a similar manner to repeated pulmonary function testing 34.

## CONCLUSIONS

^129^Xe CSSR has been demonstrated to be a reproducible method for noninvasive quantification of pulmonary microstructure and function through estimation of the alveolar septal thickness. The ST coefficient of variation was of a similar order to the variability of conventional pulmonary function tests. Furthermore, the corresponding standard deviation was less than the difference in ST between healthy volunteers and COPD patients measured here, and the volunteers and IPF patients measured previously. In future studies, the sensitivity of the technique to antifibrotic treatment response, or early changes in ILD, should be assessed to facilitate clinical application. At present, the CSSR‐derived alveolar surface‐area‐to‐volume ratio is not sufficiently reproducible for consideration as a robust clinical biomarker.

## Supporting information

Additional Supporting Information may be found in the online version of this article

Supporting Information

**Table S1**. Literature Constants Employed in ^129^Xe CSSR Studies in Human Subjects to Date

## References

[1] Kauczor HU , Surkau R , Roberts T . MRI using hyperpolarized noble gases. Eur Radiol 1998;8:820–827. 9601972 10.1007/s003300050479

[2] Mugler JP, III , Altes TA . Hyperpolarized 129Xe MRI of the human lung. J Magn Reson Imaging 2013;37:313–331. 23355432 10.1002/jmri.23844 PMC3558952

[3] van Beek EJR , Wild JM , Kauczor H‐U , Schreiber W , Mugler JP , de Lange EE . Functional MRI of the lung using hyperpolarized 3‐helium gas. J Magn Reson Imaging 2004;20:540–554. 15390146 10.1002/jmri.20154

[4] Cleveland ZI , Cofer GP , Metz G , et al. Hyperpolarized 129Xe MR imaging of alveolar gas uptake in humans. PLoS ONE 2010;5:e12192. 20808950 10.1371/journal.pone.0012192 PMC2922382

[5] Mugler JP, III , Altes TA , Ruset IC , Dregely IM , Mata JF , Miller GW , Ketel S , Ketel J , Hersman FW , Ruppert K . Simultaneous magnetic resonance imaging of ventilation distribution and gas uptake in the human lung using hyperpolarized xenon‐129. Proc Natl Acad Sci 2010;107:21707–21712. 21098267 10.1073/pnas.1011912107 PMC3003026

[6] Qing K , Ruppert K , Jiang Y , et al. Regional mapping of gas uptake by blood and tissue in the human lung using hyperpolarized xenon‐129 MRI. J Magn Reson Imaging 2014;39:346–359. 23681559 10.1002/jmri.24181 PMC3758375

[7] Butler JP , Mair RW , Hoffmann D , Hrovat MI , Rogers RA , Topulos GP , Walsworth RL , Patz S . Measuring surface‐area‐to‐volume ratios in soft porous materials using laser‐polarized xenon interphase exchange nuclear magnetic resonance. J Phys Condensed Matter 2002;14:L297–L304. 12741395 10.1088/0953-8984/14/13/103 PMC2194751

[8] Patz S , Muradyan I , Hrovat M , Dabaghyan M , Washko G , Hatabu H , Butler JP . Diffusion of hyperpolarized 129 Xe in the lung: a simplified model of 129 Xe septal uptake and experimental results. New J Phys 2011;13:015009.

[9] Ruppert K , Altes TA , Mata JF , Ruset IC , Hersman FW , Mugler JP . Detecting pulmonary capillary blood pulsations using hyperpolarized xenon‐129 chemical shift saturation recovery (CSSR) MR spectroscopy. Magn Reson Med 2016;75:1771–1780. 26017009 10.1002/mrm.25794 PMC6154503

[10] Stewart NJ , Leung G , Norquay G , et al. Experimental validation of the hyperpolarized 129Xe chemical shift saturation recovery technique in healthy volunteers and subjects with interstitial lung disease. Magn Reson Med 2015;74:196–207. 10.1002/mrm.25400 25106025

[11] Qing K , Mugler JP , Altes TA , Jiang Y , Mata JF , Miller GW , Ruset IC , Hersman FW , Ruppert K . Assessment of lung function in asthma and COPD using hyperpolarized 129Xe chemical shift saturation recovery spectroscopy and dissolved‐phase MRI. NMR Biomed 2014;27:1490–1501. 25146558 10.1002/nbm.3179 PMC4233004

[12] Galbraith SM , Lodge MA , Taylor NJ , Rustin GJS , Bentzen S , Stirling JJ , Padhani AR . Reproducibility of dynamic contrast‐enhanced MRI in human muscle and tumours: comparison of quantitative and semi‐quantitative analysis. NMR Biomed 2002;15:132–142. 11870909 10.1002/nbm.731

[13] Saam BT , Yablonskiy DA , Kodibagkar VD , Leawoods JC , Gierada DS , Cooper JD , Lefrak SS , Conradi MS . MR imaging of diffusion of 3He gas in healthy and diseased lungs. Magn Reson Med 2000;44:174–179. 10918314 10.1002/1522-2594(200008)44:2<174::aid-mrm2>3.0.co;2-4

[14] Diaz S , Casselbrant I , Piitulainen E , Pettersson G , Magnusson P , Peterson B , Wollmer P , Leander P , Ekberg O , Akeson P . Hyperpolarized 3He apparent diffusion coefficient MRI of the lung: reproducibility and volume dependency in healthy volunteers and patients with emphysema. J Magn Reson Imaging 2008;27:763–770. 18344208 10.1002/jmri.21212

[15] Morbach AE , Gast KK , Schmiedeskamp J , Dahmen A , Herweling A , Heussel CP , Kauczor HU , Schreiber WG . Diffusion‐weighted MRI of the lung with hyperpolarized helium‐3: a study of reproducibility. J Magn Reson Imaging 2005;21:765–774. 15906344 10.1002/jmri.20300

[16] Leung G , Norquay G , Schulte RF , Wild JM . Radiofrequency pulse design for the selective excitation of dissolved 129Xe. Magn Reson Med 2015;73:21–30. 24395490 10.1002/mrm.25089

[17] Chang YV , Quirk JD , Ruset IC , Atkinson JJ , Hersman FW , Woods JC . Quantification of human lung structure and physiology using hyperpolarized 129Xe. Magn Reson Med 2014;71:339–344. 24155277 10.1002/mrm.24992

[18] Norquay G , Parnell SR , Xu X , Parra‐Robles J , Wild JM . Optimized production of hyperpolarized 129Xe at 2 bars for in vivo lung magnetic resonance imaging. J Appl Phys 2013;113:044908–044909.

[19] Chang YV . MOXE: a model of gas exchange for hyperpolarized 129Xe magnetic resonance of the lung. Magn Reson Med 2013;69:884–890. 22565296 10.1002/mrm.24304

[20] Pellegrino R , Viegi G , Brusasco V , et al. Interpretative strategies for lung function tests. Eur Resp J 2005;26:948–968. 10.1183/09031936.05.00035205 16264058

[21] Bland JM , Altman DG . Measuring agreement in method comparison studies. Stat Methods Med Res 1999;8:135–160. 10501650 10.1177/096228029900800204

[22] Hajari AJ , Yablonskiy DA , Sukstanskii AL , Quirk JD , Conradi MS , Woods JC . Morphometric changes in the human pulmonary acinus during inflation. J Appl Physiol 2012;112:937–943. 22096115 10.1152/japplphysiol.00768.2011 PMC3311655

[23] Patz S , Muradian I , Hrovat MI , Ruset IC , Topulos G , Covrig SD , Frederick E , Hatabu H , Hersman FW , Butler JP . Human pulmonary imaging and Spectroscopy with hyperpolarized 129Xe at 0.2T. Acad Radiol 2008;15:713–727. 18486008 10.1016/j.acra.2008.01.008 PMC2475597

[24] Kaushik SS , Freeman MS , Yoon SW , Liljeroth MG , Stiles JV , Roos JE , Michael Foster WS , Rackley CR , McAdams HP , Driehuys B . Measuring diffusion limitation with a perfusion‐limited gas—hyperpolarized 129Xe gas‐transfer spectroscopy in patients with idiopathic pulmonary fibrosis. J Appl Physiol 2014;117:577–585. 25038105 10.1152/japplphysiol.00326.2014 PMC4157168

[25] Gläser S , Meyer R , Opitz CF , Hetzer R , Ewert R . Pulmonary interstitial and vascular abnormalities following cardiac transplantation. Transplant Proc 2008;40:3585–3589. 19100444 10.1016/j.transproceed.2008.06.074

[26] Gil J , Marchevsky AM , Jeanty H . Septal thickness in human lungs assessed by computerized interactive morphometry. Lab Invest J Tech Methods Pathol 1988;58:466–472. 3357335

[27] Kohlhase C , Maxeiner H . Morphometric investigation of emphysema aquosum in the elderly. Forensic Sci Int 2003;134:93–98. 12850401 10.1016/s0379-0738(03)00136-1

[28] Coxson HO , Rogers RM , Whittall KP , D'Yachkova Y , ParÉ PD , Sciurba FC , Hogg JC . A quantification of the lung surface area in emphysema using computed tomography. Am J Resp Crit Care Med 1999;159:851–856. 10051262 10.1164/ajrccm.159.3.9805067

[29] Yablonskiy DA , Sukstanskii AL , Woods JC , Gierada DS , Quirk JD , Hogg JC , Cooper JD , Conradi MS . Quantification of lung microstructure with hyperpolarized 3He diffusion MRI. J Appl Physiol 2009;107:1258–1265. 19661452 10.1152/japplphysiol.00386.2009 PMC2763839

[30] Driehuys B , Cofer GP , Pollaro J , Mackel JB , Hedlund LW , Johnson GA . Imaging alveolar‐capillary gas transfer using hyperpolarized 129Xe MRI. Proc Natl Acad Sci U S A 2006;103:18278–18283. 17101964 10.1073/pnas.0608458103 PMC1838742

[31] Månsson S , Wolber J , Driehuys B , Wollmer P , Golman K . Characterization of diffusing capacity and perfusion of the rat lung in a lipopolysaccaride disease model using hyperpolarized 129Xe. Magn Reson Med 2003;50:1170–1179. 14648564 10.1002/mrm.10649

[32] Hathaway EH , Tashkin DP , Simmons MS . Intraindividual variability in serial measurements of DlCO and alveolar volume over one year in eight healthy subjects using three independent measuring systems. Am Rev Resp Dis 1989;140:1818–1822. 2604306 10.1164/ajrccm/140.6.1818

[33] MacIntyre N , Crapo RO , Viegi G , et al. Standardisation of the single‐breath determination of carbon monoxide uptake in the lung. Eur Resp J 2005;26:720–735. 10.1183/09031936.05.00034905 16204605

[34] Miller MR , Hankinson J , Brusasco V , Burgos F , Casaburi R , Coates A , Crapo R , Enright P , Van der Grinten C , Gustafsson P . Standardisation of spirometry. Eur Resp J 2005;26:319–338. 10.1183/09031936.05.00034805 16055882

