# Supplementary material for: Reproducibility of quantitative indices of lung function and microstructure from 129Xe chemical shift saturation recovery (CSSR) MR spectroscopy
Source: Magn Reson Med. 2016 Jul 1;77(6):2107–13. doi: 10.1002/mrm.26310 (PMC5484314; doi:10.1002/mrm.26310)
Supplement: Supplementary file 1 — Supporting Information [file MRM-77-2107-s001.docx]

**Supplementary Material for “Reproducibility of Quantitative Indices of Lung Function and Microstructure from ^129^Xe Chemical Shift Saturation Recovery (CSSR) MR Spectroscopy”, N. J. Stewart et al.**

A: Review of literature values of xenon diffusion constants and solubilities used in ^129^Xe CSSR studies with human subjects

In the existing analytical diffusion models for estimating lung microstructural parameters from ^129^Xe CSSR data ([1-4](#_ENREF_1)), the estimate of septal thickness is directly proportional to the square root of the xenon diffusion coefficient (D) in human lung parenchymal tissue and blood, and the estimate of surface-area-to-volume ratio is inversely related to the septal thickness and the xenon solubility in the lung parenchyma (λ). The first part of this review summarizes the discrepancies in the choice of values adopted for the xenon diffusion constant and solubility in previous ^129^Xe CSSR studies in human subjects.

The diffusion coefficient in human lung tissue used in the present work was D=3.0 x 10^-6^ cm^2^s^-1^, which was taken from studies by Patz et al ([4](#_ENREF_4),[5](#_ENREF_5)). The values quoted by Patz et al were assumed to be valid, however, on further inspection, we noticed that Patz et al. cited Sta Maria and Eckmann ([6](#_ENREF_6)) as the origin of this value. In Table 1 of ([6](#_ENREF_6)), estimated diffusivities of xenon in water, blood, and brain tissue of 1.55 x 10^-5^ cm^2^s^-1^, 1.35 x 10^-5^ cm^2^s^-1^ and 5.4 x 10^-6^ cm^2^s^-1^, respectively, are quoted, which were estimated using the methods described in the appendix of that article. The closest value that we can find to 3.0x10^-6^ cm^2^s^-1^ in ([6](#_ENREF_6)) is 3.8x10^-6^ cm^2^s^-1^, which is stated as the diffusion coefficient for xenon in rat liver tissue, from ([7](#_ENREF_7)). Thus, we are unsure of the origin of the 3.0x10^-6^ cm^2^s^-1^ estimate in ([4](#_ENREF_4),[5](#_ENREF_5)); it may be that Patz et al applied some of the methods described in ([6](#_ENREF_6)) to calculate a diffusivity for xenon in lung tissue, or the value may arise from inaccurate rounding of the value from ([7](#_ENREF_7)).

Other previous ^129^Xe CSSR studies in humans ([1](#_ENREF_1),[8](#_ENREF_8),[9](#_ENREF_9)) quote a value of total dissolved-phase xenon diffusion coefficient of D=3.3x10^-6^ cm^2^s^-1^. In addition, this value has been used by other (non-CSSR-based) ^129^Xe dissolved-phase MRI studies, e.g. Dregely et al ([10](#_ENREF_10)) and Cleveland et al ([11](#_ENREF_11)). Each of these articles cites a study by Ruppert et al ([12](#_ENREF_12)) as the source of this value.

Although this value has been adopted in a number of different studies, we believe that the origins of this value and its applicability to human lung tissue should be considered. Ruppert et al ([12](#_ENREF_12)) derived this value from a ^129^Xe CSSR-type experiment in rabbits, by fitting the total dissolved-phase ^129^Xe signal intensity as a function of time post-saturation, and assuming a value for the whole-lung average septal thickness in rabbits in order to derive D. (This is essentially the reverse of what has been done in most standard ^129^Xe CSSR experiments, which is to assume a dissolved-phase xenon diffusion coefficient in order to calculate a septal thickness.)

A key limitation of the validity of this value as a standard for D is that it was derived by assuming a global mean septal thickness value in rabbits rather than measuring it by other means. Ruppert et al quote a value of 5.5 µm as the average “membrane thickness” which they use to calculate D, citing Kovar et al ([13](#_ENREF_13)) as the origin. In fact, this value is not quoted explicitly in ([13](#_ENREF_13)), so we must assume that Ruppert et al have taken this value from Figure 3c of ([13](#_ENREF_13)), which depicts septal wall thickness as a function of rabbit age. Unfortunately, Ruppert et al. do not specify the age of the rabbits used in their study, so it is not possible to verify that the assumed value of alveolar septal thickness is indeed determinable from this figure; instead, we must observe Figure 3c of ([13](#_ENREF_13)) and assume that the age of rabbits in Ruppert et al was approximately 20 weeks. (It is worth noting that Figure 3c of ([13](#_ENREF_13)) has considerable uncertainty associated with the wall thickness values (e.g. error bars ~ 0.5 µm).)

Ruppert et al ([12](#_ENREF_12)) acknowledged that there is a wide range of existing literature values for the xenon diffusion coefficient in different tissues and species, although in actuality, the extent of relevant literature is quite limited. Ruppert et al cite ([14](#_ENREF_14)) as a reference that summarizes the xenon diffusion coefficient in dog and sheep tissue samples, in addition to human blood plasma. Reproducing the values in that article (measured at 37 ºC): in water, D ~ 1.9x10^-5^ cm^2^s^-1^; in blood serum D = 1.61x10^-5^ cm^2^s^-1^ and blood plasma D = 1.51x10^-5^ cm^2^s^-1^; in sheep liver D = 2.9x10^-6^ cm^2^s^-1^; in dog muscle, D = 6.8x10^-6^ cm^2^s^-1^. Thus, it may indeed be expected that there is a wide range of xenon diffusion coefficients in different tissue types with different compositions and functions.

On a related note, it cannot be assumed that the value of D in blood plasma is identical to that of whole blood – the presence of red blood cells would be expected to cause a significantly alteration of xenon’s diffusion properties in whole blood. Hence, even if we assume that the diffusion coefficient of xenon in plasma and lung parenchyma is the same, it is difficult to infer a value of xenon diffusion coefficient in lung tissues and whole blood from the above studies, and moreover, it is likely not appropriate to assume that the diffusion coefficient is the same in tissues and whole blood; an assumption which currently underpins the ^129^Xe CSSR experiment.

Fundamentally, although Ruppert et al’s (D=3.3 x 10^-6^ cm^2^s^-1^) value appears to be the most commonly used to date, it is founded upon several key assumptions:

i) the age of the rabbits in ([12](#_ENREF_12)), ii) the accuracy of the mean thickness values in ([13](#_ENREF_13)), iii) the translatability of the situation in rabbit lungs to that in humans, iv) the equality of the diffusion coefficient in lung tissue and whole blood.

For completeness, point ii) can be reviewed further, because importantly, if the assumed tissue thickness in ([12](#_ENREF_12)) were slightly different, the value of D would be altered to a non-negligible degree. There is a paucity of literature reference values for rabbit septal tissue thickness, although as an example, here we quote two values that suggest that the mean septal thicknesses could indeed be different to the values reported in ([13](#_ENREF_13)): (i) in the same sub-species of rabbits, Mataloun et al ([15](#_ENREF_15)) determined a mean value of 9.6 ± 2.5 µm in 17 control rabbits on a normal diet and room air at 7 days old, which is larger than the value quoted in ([13](#_ENREF_13)) (approximately 7.5 µm) for rabbits of the same age; (ii) also for the same breed of rabbits, we can estimate the septal thickness from Birks et al ([16](#_ENREF_16)) by using the mean radius of curvature of capillaries and blood-gas barrier thickness reported in that work – if we assume that the septal thickness is ≈ (2 x capillary radius) + (2 x blood gas barrier thickness), we obtain (2 x 3.63) + (2 x 0.54) ≈ 8.34 µm. (However, unfortunately the ages of the rabbits are not provided in ([16](#_ENREF_16)) and hence this value cannot be directly compared with those in ([13](#_ENREF_13)).)

Finally, although not a study involving human subjects, Månsson et al ([3](#_ENREF_3)), who presented one of the first models with which to analyse ^129^Xe CSSR data, used a significantly different value of D (D = 1x10^-5^ cm^2^s^-1^) for their study in rats. This value is sourced from Wolber et al ([17](#_ENREF_17)), in which it is stated that this is the measured diffusion coefficient of xenon in plasma, in vitro. Although Ruppert et al’s value was derived at 37 ºC, there is no mention of the temperature at which the diffusion coefficient of 1x10^-5^ cm^2^s^-1^ was derived in Wolber et al’s ISMRM abstract. From ([17](#_ENREF_17)), we are led to assume that the methods used were the same as those in a previous paper by that group ([18](#_ENREF_18)), in which values for the xenon diffusion coefficient in water were obtained, at 25 º C. Thus, we can conclude that the value of 1x10^-5^ cm^2^s^-1^ is likely less appropriate for use in human ^129^Xe CSSR studies than D=3.3 x 10^-6^ cm^2^s^-1^.

In spite of the above discrepancies between studies, we can conclude that the value of D=3.3 x 10^-6^ cm^2^s^-1^ from Ruppert et al ([12](#_ENREF_12)) seems to have been generally accepted by the dissolved ^129^Xe MRI field, and going forward it may be appropriate to utilize this value until a better estimate becomes available from new measurements. However, the limitations of validity of this value should be considered in future dissolved-phase ^129^Xe NMR studies, and further work should be done in attempt to determine a more applicable estimate for human lung tissue.

--------------------------------------

There is also some inconsistency in the choice of literature values of xenon solubility in different dissolved compartments (including lung tissue) used in CSSR analysis. For example, Chang et al ([8](#_ENREF_8)) use a solubility of xenon in tissue of 0.2, citing ([19](#_ENREF_19)), whilst Patz et al ([4](#_ENREF_4),[5](#_ENREF_5)), use a solubility of xenon in lung tissue of 0.1, citing ([20](#_ENREF_20)). The value of xenon solubility in lung tissue used by Qing et al ([9](#_ENREF_9)) is not quoted in their paper.

On confirming the source of the values used by Chang et al, we cannot find a quotation of the xenon solubility in (any form of) tissue in ([19](#_ENREF_19)). Furthermore, in Chang’s earlier article ([1](#_ENREF_1)), ([19](#_ENREF_19)) is also cited, however it appears that ([13](#_ENREF_13)) might also be cited for a value of xenon solubility in tissue in the Figure 4 caption of ([1](#_ENREF_1)). There is no mention of a tissue solubility in ([13](#_ENREF_13)). Thus, we are unsure as to Chang’s motivations and evidence for using a xenon tissue solubility of 0.2, but we note that in ([1](#_ENREF_1)), the discussion states “the value we used here (λ = 0.2) was only a highly crude approximation. Approximations of the same nature apply to a few other parameters including the diffusion coefficient D of dissolved xenon.”

Although there seems to be “no universally agreed value for λ” (another quote from ([1](#_ENREF_1))), we might speculate that Patz et al.’s estimate of 0.1 is more applicable than Chang’s approximation of 0.2. Eger and Larson ([20](#_ENREF_20)) (cited by Patz et al ([4](#_ENREF_4),[5](#_ENREF_5))) quote values of the xenon water/gas partition coefficient (which is equivalent to the Ostwald solubility of xenon in water if the gas is assumed to be at a pressure of 1 atmosphere) of approximately 0.1 at 37 º C. Although ([20](#_ENREF_20)) refers to unpublished data as the source of this value, we assume that Patz et al. considered that parenchymal tissue is predominantly comprise of water, and thus that the value of 0.1 could be directly translated to that of lung tissue.

Further support for using a value of 0.1 is provided by other (non-CSSR) studies in the dissolved ^129^Xe MRI field; for example Dregely et al ([10](#_ENREF_10)), who quote that the Ostwald solubility of xenon in tissue is approximately equivalent to the blood-gas partition coefficient, which is quoted as ~ 0.1, citing ([21](#_ENREF_21)), a study which reported a value of 0.115 for the blood-gas partition coefficient of xenon at 37 ºC. In addition, it is worth noting that Cleveland et al ([11](#_ENREF_11)) cite Weathersby and Homer ([22](#_ENREF_22)) as a reference providing values for the solubility of xenon in various tissues; in ([22](#_ENREF_22)), a summary of xenon Ostwald solubilities and partition coefficients in different media is presented, and, whilst this article does not explicitly report a value of the xenon Ostwald solubility in lung tissue, we can take the quoted values of Ostwald solubility in water and blood plasma as a guideline, which suggest a value in the range 0.09 – 0.11.

--------------------------------------

Finally, although not critical to the present study (since no data about derived values of pulmonary hematocrit or blood-gas barrier thickness is presented), we review the previously-used values of the Ostwald solubility of xenon in plasma and red blood cells (RBCs). In Chang et al ([1](#_ENREF_1),[8](#_ENREF_8)), values of 0.091 and 0.19 are used for the xenon solubility in plasma and RBCs, respectively, citing ([19](#_ENREF_19)), which requires the assumption that the plasma and RBCs are saturated with air at 37 ºC. Although we accept the methodology of ([19](#_ENREF_19)) as valid, we note that it may be more appropriate to use values from Chen et al ([23](#_ENREF_23)) instead; 0.0939 and 0.271 for xenon solubility in plasma and RBCs, respectively. These values are more up-to-date than those of ([19](#_ENREF_19)), were determined in a thorough, systematic manner for a number of experimental conditions, and have been used previously by other groups in the hyperpolarized ^129^Xe MRI field, e.g. ([11](#_ENREF_11)).

--------------------------------------

A summary of the above discussion is presented in Supporting Table S1.

B: Review of literature values of ^129^Xe CSSR-derived septal thickness and surface-area-to-volume ratio values in previous studies involving human subjects

In light of the discrepancies in the values of literature constants between previous ^129^Xe CSSR studies as discussed above, the validity of comparing ^129^Xe CSSR-derived septal thickness and surface-area-to-volume ratio values between different studies is somewhat limited. Firstly, this review presents values of septal thickness and surface-area-to-volume ratio in human subjects determined from methods *other than* ^129^Xe CSSR. Secondly, we discuss the ^129^Xe CSSR-derived septal thickness and surface-area-to-volume ratio values reported in previous ^129^Xe CSSR studies in human subjects, and the appropriateness of comparing these values between different studies. The following review comprises references for morphological parameters of *human* lungs only – there are a number of studies that report these values in animal lungs, however we do not believe that these values are directly translatable to humans (see e.g. ([24](#_ENREF_24))).

(i) *ST and S/V values derived from other methods:*

A limited number of references are available that quote septal thickness values in humans (where the septal thickness has the same meaning as the parameter derived from ^129^Xe CSSR):

Gil et al ([25](#_ENREF_25)) reported septal thickness measurements by interactive computerized morphometry (using an intercept-based method with real-time video of lung samples). Assessing the range of values from a number of measurements and various experimental procedures, the septal thickness measurements presented in ([25](#_ENREF_25)) tend to lie between 8.5 and 11 µm.

Kohlhase and Maxeiner ([26](#_ENREF_26)) used the methods introduced in ([25](#_ENREF_25)) in order to assess septal thickness in elderly subjects with senile emphysema (aged 71-88 years) who had died either by drowning or cardiac complications. (Note: subjects with serious pathological findings were excluded.) Mean septal thickness values were found to be 10.5 µm and 11.1 µm; 11.3 µm and 11.3 µm in the left and right lungs of drowned and non-drowned subjects, respectively.

S. Gläser et al ([27](#_ENREF_27)) obtained lung specimens from subjects who had died some time after a heart transplantation. (Note, patients had no pulmonary complications and a mean age of 52 ± 14 years.) The average septal wall thickness for 73 patients was 9.9 ± 4.2 µm, although it is worth noting that the authors of ([27](#_ENREF_27)) suggest that these values are increased compared with what they consider to be “healthy control” values.

Although the range of literature values for septal thickness is quite large, it can concluded from the above that values of the order of 10 µm should be expected in normal subjects. We may then expect that younger subjects would exhibit a lower mean septal thickness, and older or diseased subjects would exhibit higher values.

In our present work, we quote a mean ST in healthy volunteers (mean age 59 years) of 11.6 ± 1.0 µm and 14.0 ± 2.7 µm in COPD patients in the reproducibility study, the former of which are comparable to the measurements of ST in elderly subjects as reported in ([26](#_ENREF_26)).

We were unable to find any literature values of septal thickness determined by methods other than ^129^Xe CSSR in subjects with lung disease to compare with our values obtained in COPD subjects. We discuss our values in relation to other ^129^Xe CSSR studies in humans in the second part of this review.

--------------------------------------

The extent of literature reporting surface-area-to-volume ratio in humans is slightly broader. Here, we note only two methods by which S/V can be measured:

The work of Coxson et al. ([28](#_ENREF_28)) stands out as a commonly-referenced report for S/V values determined from fixed human histology samples. This paper presents S/V values of 256 ± 24 cm^2^ml^-1^ in lungs extracted from healthy control subjects, 165 ± 23 cm^2^ml^-1^ in the lungs of mild emphysema patients, 43 ± 6 cm^2^ml^-1^ in the lungs of severe emphysema patients. (Note: 1 cm^2^ml^-1^ ≡ 1 cm^-1^.)

Also, the S/V of alveoli can be estimated from diffusion-weighted MRI of ^3^He or ^129^Xe in the gas-phase, subject to the constraints of the measured diffusion regime. An example of the derivation of S/V values from ^3^He diffusion-weighted MRI data is described in Yablonskiy et al. ([29](#_ENREF_29)), who reported the following average results for healthy volunteers and emphysema patients: S/V = 200-240 cm^-1^ in healthy control subjects, 100-140 cm^-1^ in mild emphysema patients, 50-55 cm^-1^ in severe emphysema patients.

In our work, we calculated mean S/V values in healthy volunteers and COPD patients of 194 ±70 cm^-1^ and 117 ± 60 cm^-1^, respectively, which are of a similar order to those reported in ([28](#_ENREF_28),[29](#_ENREF_29)). We can therefore postulate that our COPD patient data reflects a S/V value of somewhere between that expected for mild and severe emphysema based on ([28](#_ENREF_28),[29](#_ENREF_29)). However, the pulmonary function test results in Table 1 of our present article suggest that emphysema was quite severe in all patients except 67(F).

(ii) *ST and S/V from previous 129Xe CSSR studies in humans:*

Qing et al ([9](#_ENREF_9)) reported median septal thickness values as follows: 7.1 ± 0.9 µm in healthy subjects; 11.9 ± 3.7 µm in COPD subjects. In comparison with our values quoted in (i) above, these values are lower in both subject groups. However there are a number of reasons why a direct comparison of our values with those of ([9](#_ENREF_9)) is challenging.

Firstly, the mean age of subjects was larger in our reproducibility study (mean age 59 years) compared with ([9](#_ENREF_9)) (mean age 26 years). We have previously demonstrated a non-negligible increase in septal thickness with age ([30](#_ENREF_30)).

Secondly, Qing et al used the model of Patz et al ([4](#_ENREF_4)) to fit CSSR data, rather than MOXE (used in our present work). Previously, we have shown slight differences in the absolute values of ST derived using the two models, with a minor bias towards larger values from MOXE (see ([30](#_ENREF_30)), Figure 6). Moreover, Qing et al fitted the Patz et al model to the tissue-plasma (T/P) peak signal only, rather than the combined dissolved-phase signal. As the Patz et al model was designed for use at low field strengths where the two dissolved-phase ^129^Xe peaks could not be distinguished, the total dissolved-phase signal intensities should be fitted with that model. We postulate that using only the T/P signal could lead to a slight underestimation of the ST.

Thirdly, the mean values in ([9](#_ENREF_9)) are quoted at TLC, compared with our mean values at FRC + 1L. If we compare Qing et al’s values with the values derived at TLC in our lung inflation level experiments, the agreement between healthy volunteer data is significantly improved. Although Qing et al did not observe a statistically-significant difference between ST values measured at TLC and 50% FVC or RV, we have found a significant difference between values derived at RV + 1L and TLC, and FRC + 1L and TLC as already presented in our paper.

Finally, Qing et al used a different value of xenon diffusion coefficient in lung tissue to that used in our work, as discussed in the first section of this review.

Qing et al do not reference any literature values of ST for comparison with their derived values.

Chang et al ([8](#_ENREF_8)) reported a mean ST value of 9.2 ± 6.5 µm in healthy volunteers with a mean age of 33.7 years, including results from scans at both 1.5 T and 3 T. Chang ([1](#_ENREF_1)) did not report fitting parameters for ST from a human data-set, but quoted a value of the “xenon exchange time” of 83 ± 7.5 ms in one previously-published data-set, which corresponds to a ST value of 16.4 µm. However, because of the discrepancy in the derived S/V values from human data-sets in ([1](#_ENREF_1)) and ([8](#_ENREF_8)) as mentioned below, we do not believe that it is valid to compare our data with that presented of ([1](#_ENREF_1)).

The mean ST values in our work are comparable to those in ([8](#_ENREF_8)), though slightly larger, as might be expected since the mean age of subjects was higher in our study. However, even though the same analysis model was used in our work and ([8](#_ENREF_8)), we note that Chang et al used a slightly different value for the diffusion coefficient.

Chang et al ([8](#_ENREF_8)) compared their ST values to a literature value of 10 µm, citing ([25](#_ENREF_25)), the results of which are discussed above.

Patz et al ([4](#_ENREF_4)) reported mean septal thickness values (see Figure 6 of ([4](#_ENREF_4))) of approximately 12.5 µm in normal subjects, and 16.5 µm and 22 µm in two patients with interstitial lung disease (we have estimated these values by reading the bar chart in Figure 6). In an earlier paper, ([5](#_ENREF_5)), Patz et al only presented S/V findings and not ST findings.

Although we have employed the MOXE approach for data analysis, the values quoted in ([4](#_ENREF_4)) may be the most appropriate from other human ^129^Xe CSSR studies for comparison with our results, because we used the same values of the fundamental constants (xenon diffusion coefficient and solubility in lung tissue) as that work. As mentioned above, we previously calculated marginally higher septal thickness values with MOXE as compared to the model of Patz et al; mean difference ≈ 0.5 µm ([30](#_ENREF_30)). Although our values in elderly healthy volunteers in the present study are only slightly different to those presented by Patz et al, we are unsure of the age of the subjects in that study, but we assume it is less than that of our reproducibility cohort (see e.g. Table 1 of ([5](#_ENREF_5)) for evidence of this assumption).

Finally, as discussed in the manuscript and below, whilst we adopted the same approach as Chang et al ([8](#_ENREF_8)) for achievement of a given inflation level prior to acquiring ^129^Xe CSSR data, Patz et al used a different procedure; in their work, a number of lung volumes relative to TLC were used, which, as we have shown, can severely impact the derived ST value. Thus, the validity of comparing absolute values between different subjects at different relative inflation levels is limited.

Patz et al ([4](#_ENREF_4)) did not compare their derived ST results with any literature values from other methods.

--------------------------------------

Qing et al did not present S/V measurements from CSSR.

Chang ([1](#_ENREF_1)) derived a S/V value of 404 cm^-1^ from fitting a previously-published ^129^Xe CSSR data-set using MOXE. However, it appears that this value was derived by assuming a septal thickness value from rabbits of 5 µm (cited as originating from ([13](#_ENREF_13))). Given that Chang et al present significantly lower S/V values in their later paper ([8](#_ENREF_8)), we may assume that the value of 404 cm^-1^ is not appropriate for comparison with our work.

Chang et al (8) reported a mean S/V of 210 ± 50 cm^-1^ in healthy subjects with a mean age of 33.7 years, which appears to have been derived from the actual measured septal thickness values rather than the assumed ST of 5 µm in ([1](#_ENREF_1)). This S/V value is similar to our present measurements (194 ± 70 cm^-1^ in healthy volunteers). However, we must re-iterate the point that we used a slightly different value of the xenon diffusion coefficient in tissue to that of Chang et al, and crucially, Chang et al employed an Ostwald solubility of xenon in tissues which was a factor of 2 higher than the value we used. Thus, it may not be appropriate to compare our values of S/V with those in ([8](#_ENREF_8)).

Chang et al ([8](#_ENREF_8)) compare their inferred values of S/V to those reported in ([28](#_ENREF_28)), stating that their values are comparable to those of that study for normal subjects.

In ([1](#_ENREF_1)), a value of 250 cm^-1^ from ([28](#_ENREF_28)) is referenced for use in computational simulations of the CSSR signal, however the result upon fitting a previously-published CSSR data set from a healthy human volunteer (404 cm^-1^) is compared to a literature value of 450 cm^-1^ instead, the origin of which is unclear to the reader.

Patz et al. ([4](#_ENREF_4),[5](#_ENREF_5)) present values at different relative lung volumes between 0.25 and 1 x TLC in each subject. We believe that it would be most appropriate to compare our values of S/V with those measured by Patz et al ([4](#_ENREF_4),[5](#_ENREF_5)) at volumes of 50% TLC (~ 120 cm^-1^ in both cases). Nevertheless, in our work, the % of TLC represented by our chosen inhalation strategy of FRC + 1L means that the relative inflation level varies between subjects and could correspond to values in a wider range (e.g. 30% to 70% of TLC). Due to the observation of changes in S/V as a function of lung inflation level presented in our current manuscript, the validity of comparison of absolute values of S/V between our work and that of Patz et al. is again limited.

Patz et al compared their inferred values of S/V to those reported in ([28](#_ENREF_28)), stating that their values are ~40% lower than those in ([28](#_ENREF_28)).

--------------------------------------

References

1. Chang YV. MOXE: A model of gas exchange for hyperpolarized 129Xe magnetic resonance of the lung. Magnetic Resonance in Medicine 2013;69(3):884-890.

2. Driehuys B, Cofer GP, Pollaro J, Mackel JB, Hedlund LW, Johnson GA. Imaging alveolar-capillary gas transfer using hyperpolarized 129Xe MRI. Proceedings of the National Academy of Sciences of the United States of America 2006;103(48):18278-18283.

3. Månsson S, Wolber J, Driehuys B, Wollmer P, Golman K. Characterization of diffusing capacity and perfusion of the rat lung in a lipopolysaccaride disease model using hyperpolarized 129Xe. Magnetic Resonance in Medicine 2003;50(6):1170-1179.

4. Patz S, Muradyan I, Hrovat M, Dabaghyan M, Washko G, Hatabu H, Butler JP. Diffusion of hyperpolarized 129 Xe in the lung: a simplified model of 129 Xe septal uptake and experimental results. New Journal of Physics 2011;13(1):015009.

5. Patz S, Muradian I, Hrovat MI, Ruset IC, Topulos G, Covrig SD, Frederick E, Hatabu H, Hersman FW, Butler JP. Human Pulmonary Imaging and Spectroscopy with Hyperpolarized 129Xe at 0.2T. Academic Radiology 2008;15(6):713-727.

6. Sta Maria N, Eckmann DM. Model predictions of gas embolism growth and reabsorption during xenon anesthesia. Anesthesiology 2003;99(3):638-645.

7. Evans AL, Busuttil A, Gillespie FC, Unsworth J. The rate of clearance of xenon from rat liver sections in vitro and its significance in relation to intracellular diffusion rates. Physics in Medicine and Biology 1974;19(3):303.

8. Chang YV, Quirk JD, Ruset IC, Atkinson JJ, Hersman FW, Woods JC. Quantification of human lung structure and physiology using hyperpolarized 129Xe. Magnetic Resonance in Medicine 2014;71(1):339-344.

9. Qing K, Mugler JP, Altes TA, Jiang Y, Mata JF, Miller GW, Ruset IC, Hersman FW, Ruppert K. Assessment of lung function in asthma and COPD using hyperpolarized 129Xe chemical shift saturation recovery spectroscopy and dissolved-phase MRI. NMR in Biomedicine 2014;27(12):1490-1501.

10. Dregely I, Mugler JP, III, Ruset IC, Altes TA, Mata JF, Miller GW, Ketel J, Ketel S, Distelbrink J, Hersman FW, Ruppert K. Hyperpolarized Xenon-129 gas-exchange imaging of lung microstructure: first case studies in subjects with obstructive lung disease. Journal of magnetic resonance imaging : JMRI 2011;33(5):1052-1062.

11. Cleveland ZI, Cofer GP, Metz G, Beaver D, Nouls J, Kaushik SS, Kraft M, Wolber J, Kelly KT, McAdams HP, Driehuys B. Hyperpolarized 129Xe MR Imaging of Alveolar Gas Uptake in Humans. PLoS ONE 2010;5(8):e12192.

12. Ruppert K, Mata JF, Brookeman JR, Hagspiel KD, Mugler JP, III. Exploring Lung Function with Hyperpolarized 129Xe Nuclear Magnetic Resonance. Magnetic Resonance in Medicine 2004;51(4):676-687.

13. Kovar J, Sly PD, Willet KE. Postnatal alveolar development of the rabbit. Journal of Applied Physiology 2002;93(2):629-635.

14. Unsworth J, Gillespie F. Diffusion coefficients of xenon and krypton in water from 0 C to 80 C and in biological tissues at 37 C. In: Diffusion Processes: Proceedings of the Thomas Grahm Memorial Symposium, London 1971:599–608.

15. Mataloun MMGB, Leone CR, Mascaretti RS, Dohlnikoff M, Rebello CM. Effect of postnatal malnutrition on hyperoxia-induced newborn lung development. Brazilian Journal of Medical and Biological Research 2009;42:606-613.

16. Birks EK, Mathieu-Costello O, Fu Z, Tyler WS, West JB. Comparative aspects of the strength of pulmonary capillaries in rabbit, dog, and horse. Respiration Physiology 1994;97(2):235-246.

17. Wolber J, Santoro D, Leach MO, Bifone A. Diffusion of Hyperpolarized 129Xe in Biological Systems: Effects of Chemical Exchange. Proceedings of the 8th Annual Meeting of the International Society for Magnetic Resonance in Medicine, Denver, Colorado 2000:Abstract 754.

18. Wolber J, Doran SJ, Leach MO, Bifone A. Measuring diffusion of xenon in solution with hyperpolarized 129 Xe NMR. Chemical Physics Letters 1998;296(3-4):391-396.

19. Ladefoged J, Andersen AM. Solubility of Xenon-133 at 37°C in water, saline, olive oil, liquid paraffin, solutions of albumin, and blood. Physics in Medicine and Biology 1967;12(3):353-358.

20. Eger EI, Larson CP. Anaesthetic Solubility In Blood And Tissues: Values And Significance. British Journal of Anaesthesia 1964;36(3):140-149.

21. Goto T, Suwa K, Uezono S, Ichinose F, Uchiyama M, Morita S. The blood-gas partition coefficient of xenon may be lower than generally accepted. British Journal of Anaesthesia 1998;80(2):255-256.

22. Weathersby PK, Homer LD. Solubility of inert gases in biological fluids and tissue: a review. Undersea Biomedical Research 1980;7(4):277-296.

23. Chen RYZ, Fan FC, Kim S. Tissue-blood partition coefficient for xenon: temperature and hematocrit dependence. Journal of Applied Physiology Respiratory Environmental and Exercise Physiology 1980;49(2):178-183.

24. Mercer RR, Russell ML, Crapo JD. Alveolar septal structure in different species. Journal of Applied Physiology 1994;77(3):1060-1066.

25. Gil J, Marchevsky AM, Jeanty H. Septal thickness in human lungs assessed by computerized interactive morphometry. Laboratory investigation; a journal of technical methods and pathology 1988;58(4):466-472.

26. Kohlhase C, Maxeiner H. Morphometric investigation of emphysema aquosum in the elderly. Forensic science international 2003;134(2–3):93-98.

27. Gläser S, Meyer R, Opitz CF, Hetzer R, Ewert R. Pulmonary Interstitial and Vascular Abnormalities Following Cardiac Transplantation. Transplantation proceedings 2008;40(10):3585-3589.

28. Coxson HO, Rogers RM, Whittall KP, D'Yachkova Y, ParÉ PD, Sciurba FC, Hogg JC. A Quantification of the Lung Surface Area in Emphysema Using Computed Tomography. American Journal of Respiratory and Critical Care Medicine 1999;159(3):851-856.

29. Yablonskiy DA, Sukstanskii AL, Woods JC, Gierada DS, Quirk JD, Hogg JC, Cooper JD, Conradi MS. Quantification of lung microstructure with hyperpolarized 3He diffusion MRI. Journal of Applied Physiology 2009;107(4):1258-1265.

30. Stewart NJ, Leung G, Norquay G, Marshall H, Parra-Robles J, Murphy PS, Schulte RF, Elliot C, Condliffe R, Griffiths PD, Kiely DG, Whyte MK, Wolber J, Wild JM. Experimental validation of the hyperpolarized 129Xe chemical shift saturation recovery technique in healthy volunteers and subjects with interstitial lung disease. Magnetic Resonance in Medicine 2015;74(1):196-207.

Tables

Supporting Table S1: Literature constants employed in ^129^Xe CSSR studies in human subjects to date
