# Supplementary material for: Reproducibility of quantitative indices of lung function and microstructure from 129Xe chemical shift saturation recovery (CSSR) MR spectroscopy
Source: Magn Reson Med. 2016 Jul 1;77(6):2107–13. doi: 10.1002/mrm.26310 (PMC5484314; doi:10.1002/mrm.26310)
Supplement: Supplementary file 2 — Table S1. Literature Constants Employed in 129Xe CSSR Studies in Human Subjects to Date [file MRM-77-2107-s002.docx]

Supporting Table S1: Literature constants employed in ^129^Xe CSSR studies in human subjects to date

| **Paper** | **D (cm^2^s^-1^)** | **λ (tissue)** | **λ_PL_** | **λ_RBC_** | **Fitting model** |
| --- | --- | --- | --- | --- | --- |
| **Patz et al (**[**1**](#_ENREF_1)**,**[**2**](#_ENREF_2)**)** | 3.0x10^-6^ | 0.1 | N/A | N/A | Patz et al (to combined dissolved-phase peak) |
| **Chang et al (**[**3**](#_ENREF_3)**)** | 3.3x10^-6^ | 0.2 | 0.091 | 0.19 | Chang (MOXE) |
| **Qing et al (**[**4**](#_ENREF_4)**)** | 3.3x10^-6^ | N/S | N/A | N/A | Patz et al (to T/P peak only) |
| **Stewart et al (**[**5**](#_ENREF_5)**)** | 3.0x10^-6^ | 0.1  (N/S) | 0.091  (N/S) | 0.19  (N/S) | Chang (MOXE), Patz et al (to combined peaks) and Månsson et al |
| **Present paper (Stewart et al)** | 3.0x10^-6^ | 0.1 | N/A | N/A | Chang (MOXE) |
| **Most up-to-date / widely-used literature values (+ source)** | 3.3x10^-6^  ([6](#_ENREF_6)) | 0.1  ([7](#_ENREF_7)) | 0.0939  ([8](#_ENREF_8)) | 0.271  ([8](#_ENREF_8)) | N/A |

N/A.: not applicable; N/S.: not specified

References

1. Patz S, Muradian I, Hrovat MI, Ruset IC, Topulos G, Covrig SD, Frederick E, Hatabu H, Hersman FW, Butler JP. Human Pulmonary Imaging and Spectroscopy with Hyperpolarized 129Xe at 0.2T. Academic Radiology 2008;15(6):713-727.

2. Patz S, Muradyan I, Hrovat M, Dabaghyan M, Washko G, Hatabu H, Butler JP. Diffusion of hyperpolarized 129 Xe in the lung: a simplified model of 129 Xe septal uptake and experimental results. New Journal of Physics 2011;13(1):015009.

3. Chang YV, Quirk JD, Ruset IC, Atkinson JJ, Hersman FW, Woods JC. Quantification of human lung structure and physiology using hyperpolarized 129Xe. Magnetic Resonance in Medicine 2014;71(1):339-344.

4. Qing K, Mugler JP, Altes TA, Jiang Y, Mata JF, Miller GW, Ruset IC, Hersman FW, Ruppert K. Assessment of lung function in asthma and COPD using hyperpolarized 129Xe chemical shift saturation recovery spectroscopy and dissolved-phase MRI. NMR in Biomedicine 2014;27(12):1490-1501.

5. Stewart NJ, Leung G, Norquay G, Marshall H, Parra-Robles J, Murphy PS, Schulte RF, Elliot C, Condliffe R, Griffiths PD, Kiely DG, Whyte MK, Wolber J, Wild JM. Experimental validation of the hyperpolarized 129Xe chemical shift saturation recovery technique in healthy volunteers and subjects with interstitial lung disease. Magnetic Resonance in Medicine 2015;74(1):196-207.

6. Ruppert K, Mata JF, Brookeman JR, Hagspiel KD, Mugler JP, III. Exploring Lung Function with Hyperpolarized 129Xe Nuclear Magnetic Resonance. Magnetic Resonance in Medicine 2004;51(4):676-687.

7. Goto T, Suwa K, Uezono S, Ichinose F, Uchiyama M, Morita S. The blood-gas partition coefficient of xenon may be lower than generally accepted. British Journal of Anaesthesia 1998;80(2):255-256.

8. Chen RYZ, Fan FC, Kim S. Tissue-blood partition coefficient for xenon: temperature and hematocrit dependence. Journal of Applied Physiology Respiratory Environmental and Exercise Physiology 1980;49(2):178-183.
